# Association between small dense low-density lipoprotein and carotid intima-media thickness

**DOI:** 10.34172/jcvtr.33145

**Published:** 2024-12-23

**Authors:** Mohsen Khosravi, Farhad Sheikhnia, Mohammad Reza Pashaei, Maryam Karimi-Dehkordi, Shahin Alizadeh-Fanalou

**Affiliations:** ^1^Department of Biochemistry, Faculty of Medicine, Baqiyatallah University of Medical Sciences, Tehran, Iran; ^2^Student Research Committee, Urmia University of Medical Sciences, Urmia, Iran; ^3^Department of Clinical Biochemistry, School of Medicine, Urmia University of Medical Sciences, Urmia, Iran; ^4^Patient Safety Research Center, Clinical Research Institute, Urmia University of Medical Sciences, Urmia, Iran; ^5^Department of Clinical Sciences, Faculty of Veterinary Medicine, Shahrekord Branch, Islamic Azad University, Shahrekord, Iran

**Keywords:** Small dense low-density lipoprotein, Carotid intima-media thickness, Atherosclerosis, Cholesterol

## Abstract

Cardiovascular disease (CVD) and atherosclerosis are major causes of mortality worldwide. Early and accurate diagnosis of vascular thickening by predictive markers can help reduce the death rate of these diseases. Low-density lipoprotein (LDL) particles, which are rich in cholesterol, are regarded as key biomarkers for CVD and atherosclerosis. Numerous studies have demonstrated that pattern B (small dense LDL, sdLDL) is more atherogenic than LDL and can serve as a superior quantitative marker for CVD than LDL. Furthermore, several studies have indicated that carotid intima-media thickness (cIMT) is a reliable marker for the early detection of CVD and atherosclerosis in various populations. This review examines the relationship between sdLDL concentration and cIMT. It is concluded that sdLDL concentration has a positive correlation with cIMT, and their combined use can facilitate a more precise assessment of the diseases, especially atherosclerosis.

## Introduction

 Metabolic diseases, especially diabetes, metabolic syndrome (MetS), cardiovascular diseases (CVD), and atherosclerosis, pose major health challenges for modern societies.^1,2^ Atherosclerosis, which has a high prevalence, has been extensively studied to elucidate its etiology and risk factors. Atheroma plaque formation is dependent on the cholesterol uptake by endothelial cells of the arterial wall from the circulation. Hence, hypercholesterolemia is a well-established risk factor for atherosclerosis. Low-density lipoprotein (LDL) particles, which are rich in cholesterol, are recognized as a key biomarker of metabolic disorders, such as atherosclerosis.^1-5^

 LDL particles are heterogeneous in the circulation and can be categorized into two patterns based on their size, density, and composition: pattern A (large buoyant LDL, lbLDL) and pattern B (small dense LDL, sdLDL). Several studies have demonstrated that sdLDL plays a role in the pathogenesis of CVD and atherosclerosis. Hence, it is hypothesized that sdLDL has a considerable diagnostic potential and can be used as a main marker for early detection of CVD and atherosclerosis.^1,2,6^

 Blood vessels consist of three layers: the tunica intima, the tunica media, and the tunica adventitia from inside to outside, respectively. Intima-media thickness (IMT), also known as intimal medial thickness, denotes the thickness of the tunica intima and media layers, which is typically assessed by a simple, noninvasive external ultrasound technique.^7^ Carotid IMT (cIMT) is reported to be correlated with CVD and the severity of atherosclerosis and can serve as a prognostic marker of cardiovascular events.^8-10^ Moreover, it increases with aging, diabetes, and chronic kidney disease.^11^ The thickening of the tunica intima and media is complex. A wide range of factors, such as blood pressure, local hemodynamics, shear stress, and circumferential tensile stress, have a significant impact on cIMT.^12^

 The objective of our review was to explore the association between sdLDL levels and cIMT in patients with various metabolic disorders, particularly atherosclerosis. Additionally, this review examines the relationship of sdLDL levels to cIMT in comparison with other lipid parameters based on existing literature. The aim is to provide a comprehensive overview of how sdLDL can improve the identification of relevant diseases more effectively and accurately.

## sdLDL and atherosclerosis

 LDL is a cholesterol-rich lipoprotein in the circulation and can be divided into two patterns based on their size, density, and composition: lbLDL and sdLDL. Plasma levels of LDL are currently regarded as a crucial risk factor for CVD, particularly atherosclerosis. Numerous studies have demonstrated that most of the detrimental and pathogenic effects of LDL particles are mainly attributed to sdLDL due to several features, such as low binding affinity to LDLR, increased penetration to the arterial wall, enhanced susceptibility to oxidation and glycation, as well as a high prevalence in individuals with metabolic diseases.^1,2^

 The pathogenesis of atherosclerosis by sdLDL is dependent on these characteristics.^13^ They prolong the half-life of sdLDL in the circulation and increase its retention to the vessel wall by binding to proteoglycans of intima.^14,15^ The retained and oxidized sdLDL in the subendothelial layers is engulfed by tissue macrophages. Subsequently, the macrophages are transformed into foam cells and plaques in the subendothelium, which is the main cause of atherosclerosis.^16-18^ Therefore, sdLDL triggers proinflammatory processes in the vessel wall, which is the initial stage of atherosclerosis. Several studies indicate that serum sdLDL is linked to many metabolic diseases, especially CVD, atherosclerosis, MetS, and diabetes, and can be used as a better marker than LDL (and other lipid parameters) for early detection of the diseases.^14,19-27^ Hence, sdLDL was termed as an atherogenic index of plasma.^28^

 As discussed in various studies, reducing sdLDL is an effective strategy for managing the diseases mentioned above. Literature indicates that various LDL-lowering drugs, such as statins, ezetimibe, fibrates, doxazocin, orlistat, niacin, and a healthy lifestyle and dietary agents, especially phytosterols and omega-3 fatty acids, and Glucose-Lowering Drugs, such as Metformin, Sodium-glucose cotransporter 2 (SGLT2) inhibitors, Glucagon-Like Peptide-1 Receptor Agonists (GLP-1RAs), Dipeptidyl peptidase-4 (DPP-4) inhibitors, Glitazones, can significantly decrease sdLDL levels and consequently lower the risk of atherosclerosis.^2^

## Carotid IMT and atherosclerosis

**Figure 1:**
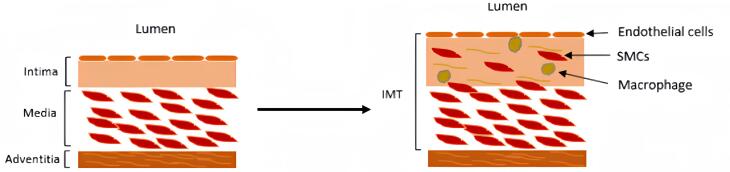
Process of intima-media thickness (IMT) in the carotid artery wall. Under these conditions, macrophages and vascular smooth muscle cells migrate to the intima layer

 cIMT is an indicator of the thickness of the tunica intima and tunica media, the two inner layers of the carotid wall (Figure 1). cIMT is assessed between the intimal-luminal and the medial-adventitial interfaces by B-mode carotid ultrasound in relatively large vessels close to the skin, mainly the carotid arteries.^7,29,30^ Alternative methodologies for the measurement of cIMT are outlined in Table1. Several studies have observed cIMT increases in patients with metabolic disorders such as diabetes, dyslipidemia, familial hypercholesterolemia, hemodialysis, ischemic stroke, and atherosclerosis.^10,25,26,28^

**Table 1:** Different methods of cIMT measurement

**Method**	**Description**	**Advantages**	**Limitations**	**Ref**
**B-Mode Ultrasound**	Provides a two-dimensional image of the carotid artery, allowing visualization of the intimal-luminal and medial-adventitial interfaces.	Non-invasive, widely available, relatively low cost.	Operator-dependent, potential for interobserver and intraobserver variability.	^31,32^
**M-Mode Ultrasound**	Provides a one-dimensional view of structures along a single ultrasound line, displaying all structures along that line over time.	Excellent temporal and axial resolution, useful for quantifying mobility of structures and measuring dimensions.	Only displays a single ultrasound line, making it difficult to obtain representative sections of structures.	^33^
**Automated Edge Detection Systems**	Uses software algorithms to automatically detect and measure cIMT from B-mode ultrasound images.	Reduces operator dependence and variability, increases measurement reproducibility.	Requires high-quality images and sophisticated software.	^34^
**Three-Dimensional (3D) Ultrasound**	Provides volumetric imaging of the carotid artery, allowing for more detailed assessment of cIMT and plaque morphology.	Provides a more comprehensive view of the carotid artery, including plaque characteristics.	More complex and time-consuming than traditional B-mode ultrasound, requires specialized equipment.	^35,36^
**Magnetic Resonance Imaging (MRI)**	Non-invasive imaging technique that can measure cIMT with high resolution and without exposure to ionizing radiation.	High-resolution images, no ionizing radiation, can assess vessel wall characteristics.	Expensive, time-consuming, and not as widely available as ultrasound.	^37^
**Computed Tomography (CT)**	High-resolution CT scans are performed to measure cIMT, particularly in research settings.	High-resolution images, can assess calcified plaques.	Exposure to ionizing radiation, expensive, less commonly used for cIMT measurement compared to ultrasound and MRI.	^37^
**Speckle Tracking Ultrasound**	Tracks movement of speckles within ultrasound image to measure arterial wall motion and deformation, providing additional information on arterial stiffness and elasticity.	Detailed insights into arterial wall dynamics, detects early changes in arterial stiffness.	More complex and time-consuming than traditional B-mode ultrasound, requires specialized equipment/expertise.	^32,38^
**Automated Measurement Systems**	Uses AI and machine learning algorithms to analyze ultrasound images and measure cIMT, processing large datasets quickly and providing consistent results.	High accuracy and reproducibility, reduced operator dependency, efficient handling of large volumes of data.	Requires advanced technology and infrastructure, may involve higher costs.	^39^

 Several factors have been reported to influence cIMT, such as hypertension, aging, smoking, body mass index, fasting blood glucose, fasting serum insulin, and cardiovascular risk factors, including high total cholesterol, high LDL, high fasting blood sugar, low high-density lipoprotein (HDL),^40-43^ fibrinogen, LOX-1 (lectin-type oxidized LDL receptor 1), hs-CRP (high-sensitivity C-reactive protein),^44^ and the LDL/HDL ratio.^45^ Some studies have also investigated the effect of sdLDL levels on cIMT.^46,47^ These factors are also recognized as risk factors for CVD and atherosclerosis.

 Several clinical trials have demonstrated that statin therapy can markedly reduce arterial cIMT.^48-50^ Furthermore, a review of randomized, controlled trials showed that long-term antihypertensive treatment has a mitigating effect on cIMT progression, irrespective of types of drugs.^51^

 Additionally, a network meta-analysis comparing the effects of lipid-lowering, hypoglycemic, antihypertensive, and antiplatelet agents on cIMT progression indicated that phosphodiesterase III inhibitors and calcium channel blockers are more efficacious than other drug classes in inhibiting cIMT progression.^52^

 A meta-analysis study revealed that the association between cIMT and the risk of subsequent cardiovascular events makes cIMT a robust predictor of vascular events. Hence, it was recommended that cIMT measurement is beneficial for evaluating the risk of CVD in patients with metabolic disorders.^53^

 However, a meta-analysis did not verify the association between cIMT changes (progression) and cardiovascular events in a large population. However, it was initially observed that cIMT is related to the risk of cardiovascular events in the general population (as a biomarker of CVD and atherosclerosis).^54^ An important point about this article should be noted. cIMT is measured using ultrasound with a linear probe, and clinicians’ experience is crucial for interpreting results and reducing the variabilities mentioned above. Nevertheless, this technique has some limitations, such as the interobserver and intraobserver variabilities.^55^

 Overall, considering the simplicity, non-invasiveness, and repeatability of arterial cIMT measurements using external ultrasound in large arteries close to the skin, it can be utilized as a surrogate marker for monitoring, regression, and the progression of CVD and atherosclerosis.^7,29,30^

## Carotid IMT and sdLDL

 At the outset of the article, it was established that sdLDL and cIMT are independently and significantly correlated with CVD and atherosclerosis based on many studies.^26^ The aim of this study was to examine the relationship between sdLDL and cIMT in individuals with CVD, particularly atherosclerosis. A cohort study on Japanese subjects with dyslipidemia, diabetes, and hypertension revealed that sdLDL concentration, measured by homogenous assay, was strongly associated with cIMT compared to other lipid parameters.^56^

 Another study measured sdLDL concentration in healthy, 50-year-old men using the gradient gel electrophoresis technique and found a strong association between sdLDL and cIMT.^57^ LDL subclasses and cIMT were assessed using a non-denaturing technique called linear polyacrylamide gel electrophoresis (Lipoprint) and Color Doppler Ultrasound, respectively. SdLDL particles, designated as LDL-3 to -7. Particularly, smallest LDL-5 were associated with the change in cIMT in cases of Moderate Hypercholesterolemia.^58^ Using a standardized protocol, we performed high resolution B-mode ultrasonography of the common carotid arteries with a linear array probe. We manually measured the IMT using a vernier caliper after taking a picture. We used non-denaturing polyacrylamide 3–31 % gradient gel electrophoresis for LDL subclass separation and size estimation. There was a significant association between LDL particle size and cIMT in CAD patients, independent of traditional lipid and established risk factors. SdLDL particles were associated with vascular changes and atherosclerotic progression. Our findings suggest that high-resolution polyacrylamide gel electrophoresis of LDL particle size distribution could be a useful method to estimate individual cardiovascular risk and atherosclerosis progression in both symptomatic and asymptomatic individuals.^59^ According to the study conducted by Shoji et al, there was a significantly stronger correlation between cIMT and serum sdLDL levels measured by simple precipitation technique in subjects with these risk factors compared to other lipid parameters.^60^ Erqou and colleagues.^61^ measured sdLDL by vertical auto profile technique in White and Black individuals and found that sdLDL had an independent correlation with increased cIMT, with this relationship exhibiting comparable patterns among both groups. Interestingly, Black individuals demonstrated higher cIMT. Lin et al^62^ investigated the association of lipoprotein profile, urinary levels of cadmium and lead, and cIMT in 736 Taiwanese subjects without lipid-lowering medication and had a mean age of 21.3 years. They used automated homogeneous methods with detergents to quantify sdLDL-C levels. They found a positive correlation between natural logarithm-transformed lead and cadmium levels and LDL-C, sdLDL-C, LDL-TG, and cIMT. cIMT increased for each unit increase in LDL-C, sdLDL-C, and apolipoprotein B. In the separate analyses, both metals were positively associated with LDL-C and sdLDL-C, and both lipoprotein parameters were positively associated with cIMT. Higher levels of these metals were associated with higher cIMT. In the joint analysis, urinary lead and cadmium increased LDL-C. However, only lead, not cadmium, increased sdLDL-C.^62^ By defining abnormal carotid IMT as IMT > 1.0 mm, and sdLDL-C as the sum of LDL3-C, LDL4-C, and LDL5-C, the abnormal IMT group showed significantly increased levels of sdLDL-C (particularly LDL3-C and LDL4-C) and significantly decreased levels of LDL1-C than the control group. Abnormal IMT was positively correlated with sdLDL-C, especially LDL3-C and LDL4-C. Subjects with carotid plaque (CAP) had significantly higher plasma sdLDL-C levels, consisting of LDL3-C, LDL4-C and LDL5-C. Interestingly, unstable CAP was more likely to occur with higher levels of sdLDL-C, and thereby, sdLDL-C was an independent predictor of CAP in subjects with abnormal IMT in the Chinese population.^63^ Another study defined sdLDL as the sum of LDL-5, LDL-6, and LDL-7, and revealed that in patients with unstable plaques, the levels of sdLDL were significantly increased compared to those found in patients with stable plaques.^64^

 The incorporation of liraglutide into metformin monotherapy resulted in a significant reduction in small dense LDL-3 and LDL-4 subfractions in patients with type 2 diabetes mellitus. Only a significant correlation was noted between alterations in cIMT and those in the sdLDL-3 subfraction, and the only independent predictor of cIMT was sdLDL-3 particles.^65^ The use of Pioglitazone-Flutamide-Metformin polytherapy increased the radii of sdLDL and large HDL lipoprotein subclasses in Polycystic Ovary Syndrome (PCOS) patients. Additionally, cIMT demonstrated a substantial decrease after 30 months of polytherapy.^66^ In another study of PCOS, however, cIMT and sdLDL were not different between PCOS-adolescents who were overweight or obese and those who had a normal body weight. Similarly, there was no significant difference in these values between PCOS-adolescents and healthy controls who were matched in terms of sex and age. Interestingly, cIMT values were significantly influenced by systolic blood pressure levels, whereas sdLDL levels were not similarly affected.^67^ Furthermore, Basu et al reported a positive correlation between cIMT and sdLDL by nuclear magnetic resonance in subjects with type 1 diabetes.^68^ Moreover, Ichikawa et al observed a significant association between sdLDL, by simple precipitation, and cIMT before treatment with direct-acting antivirals in subjects infected with hepatitis-C virus.^46^ Shen et al noted a strong and positive association between serum sdLDL concentrations by homogenous assay and cIMT in healthy Chinese subjects (Table 2).^47^

**Table 2:** The relationship between sdLDL and cIMT

**Measurement Method **		**Participants**	**Finding(s)**	**Ref**
**cIMT**	**SdLDL**			
Ultrasound (10 mHz direct probe)	Quantimetrix lipoprint system LDL subfraction kit	54 ischemic stroke patients vs 50 controls	A positive correlation between right/left cIMT and sd-LDL	^26^
Ultrasonography (7.5 MHz high-resolution probe)	-	31 hemodialysis patients vs 31 healthy subjects	A significant correlation between atherogenic index of plasma (the functional marker of the LDL particular size) and cIMT	^28^
High-resolution color Doppler ultrasound (7.5 MHz annular array probe)	Quantimetrix LipoPrint system	183 healthy individuals	A strong correlation between sdLDL-C concentrations and cIMT	^47^
Ultrasonography (7.5-MHz linear type B-mode probe)	Homogenous assay	97 individuals	A positive correlation between sdLDL-C and sdLDL	^56^
Ultrasound (8-MHz high-resolution annular array scanner)	density gradient ultracentrifugation (DGUC) and nondenaturing polyacrylamide gradient gel electrophoresis (GGE)	94 healthy subjects	A strong correlation between Common Carotid Artery IMT and sdLDL particle subfraction (LDL-III)	^57^
High resolution B-mode ultrasonography (linear array probe (7.5 MHz))	Non-denaturing polyacrylamide 3–31% gradient gel electrophoresis (PAGE)	100 patients with coronary heart disease vs 100 healthy controls	An inverse correlation between the IMT of the carotid arteries and the size of LDL	^69^
B mode ultrasound (linear 10 MHz Probe)	-	36 subclinical hypothyroidism individuals vs 64 euthyroid subjects	A positive correlation between mean IMT values in both common carotid arteries and sdLDL	^70^
High-resolution ultrasound	Quantimetrix Lipoprint LDL system	368 acute ischaemic stroke patients vs 165 non acute ischaemic stroke controls	IMT displayed a positive correlation with the concentrations of sdLDL-C. SdLDL-C emerged as an independent risk factor for IMT.	^71^
B-mode ultrasonography (10 MHz probe)	Homogeneous assay	2030 individuals free of cardiovascular disease	sdLDL-C significantly and positively correlated with the progression of cIMT.	^72^
Ultrasonography	Peroxidase assay	471 patients with carotid atherosclerosis vs 1107 subjects with non- carotid atherosclerosis	The serum sdLDL-C has been identified as an independent determinant and valuable risk indicator of carotid atherosclerosis.	^73^
Ultrasonography (7.5- to 12-MHz linear-array transducer and a duplex scanner)	Quantimetrix Lipoprint LDL system	50 patients with Psoriatic arthritis (PsA) vs 100 controls	A positive correlation between the LDL score (%sdLDL) and cIMT, a negative correlation between LDL diameter and cIMT in patients with PsA.	^74^
High-resolution ultrasound (5 to 12-MHz multi-frequency linear array probe)	-	98 patients with type 2 diabetes mellitus (T2DM)	The TG/HDLC ratio, an indicator of sdLDL particles, has the potential to serve as a straightforward and beneficial instrument for forecasting the augmented cIMT in Chinese adolescents and young adults who have recently been diagnosed with T2DM.	^75^
High-resolution B-mode ultrasound	Lipoprint® electrophoresis (Quantimetrix)	100 MetS patients vs 50 healthy controls	A connection between certain lipoprotein subfractions and the atherogenicity that was directly assessed from cIMT was not observed	^76^
B-mode ultrasound	Lipoprint System	228 post-menopausal women	A significant association between CIMT and mean LDL particle size, as well as LDL score (a measure of sd-LDL)	^77^
High-resolution B-mode ultrasound (9-3 MHz linear array transducer)	Nondenaturing polyacrylamide gradient gel electrophoresis	59 patients with (pre)diabetes	A potential association between elevated levels of sdLDL particles and an augmentation in cIMT	^78^
High-resolution B-mode carotid ultrasonography	Proton nuclear magnetic resonance (NMR) spectroscopy)	6572 participants in the Multi-Ethnic Study of Atherosclerosis (MESA)	A constant and direct association between sdLDL-C and cIMT throughout different eGFR levels.	^79^

 According to the studies mentioned above, sdLDL concentration is strongly associated with cIMT measurement in various patients and healthy individuals. Therefore, sdLDL can act as a useful predictor of cIMT, and an efficient marker for early diagnosis of metabolic diseases and CVD, particularly atherosclerosis. Moreover, sdLDL, as a risk marker of CVD and atherosclerosis, is more closely related to cIMT than other lipid parameters.^25,26,28^ Furthermore, sdLDL is strongly correlated with insulin resistance and glucose tolerance, supporting its potential as a valuable biomarker in evaluating cIMT and the current extent of atherosclerosis.^80^

 However, in different studies, sdLDL concentrations are measured by various methods. Therefore, differences in measurement methods are one of the reasons for the variation in the association between cIMT and sdLDL in independent studies.

 These studies have been performed independently in various conditions, including hypertension, smoking, dyslipidemia, MetS, atherosclerosis, hemodialysis,^28^ familial hyperlipidemia, and *type 1 *and *2 *diabetes.^56,57,60,68^

## Discussion

 The relationship between sdLDL concentration and cIMT measurement has been extensively studied across various populations, including individuals with different metabolic disorders, ethnic backgrounds, and age groups. This diversity is crucial for understanding the generalizability and applicability of sdLDL as a biomarker for CVD and atherosclerosis.

## Implications of Different Populations

###  Metabolic disorders

 Studies have shown that sdLDL concentration is strongly associated with cIMT in individuals with metabolic disorders such as diabetes, dyslipidemia, and metabolic syndrome. For example, in patients with type 2 diabetes, sdLDL levels were found to be a significant predictor of cIMT, indicating a higher risk of atherosclerosis in this population. Similarly, in individuals with familial hypercholesterolemia, elevated sdLDL levels were correlated with increased cIMT.^10,25,26,28^

###  Ethnic backgrounds

 The relationship between sdLDL and cIMT has also been investigated in different ethnic groups. For instance, a study comparing White and Black individuals found that sdLDL had an independent correlation with increased cIMT in both groups, although Black individuals demonstrated higher cIMT values. This suggests that while sdLDL is a consistent marker across ethnicities, there may be additional factors influencing cIMT in different populations.^61^

###  Age groups

 Age is another important factor influencing the relationship between sdLDL and cIMT. Studies have shown that sdLDL levels and cIMT increase with age, and the association between these two markers is more pronounced in older adults. This highlights the importance of considering age when evaluating sdLDL and cIMT as risk markers for CVD.^72^

 The differences observed between these diverse populations underscore the need for tailored approaches in clinical practice. For example, the stronger correlation between sdLDL and cIMT in individuals with metabolic disorders suggests that sdLDL could be a more valuable marker for early detection and risk assessment in these patients. Additionally, the variations in cIMT values among different ethnic groups indicate that population-specific reference ranges may be necessary for accurate risk stratification.

## Conclusion

 In conclusion, cIMT can be considered as a surrogate marker of CVD and atherosclerosis. Due to the positive correlation between sdLDL and cIMT, measuring plasma sdLDL levels is useful for early diagnosis of subclinical diseases, than other lipid parameters.

## Competing Interests

 The authors declare that there are no conflicts of interest

## Ethical Approval

 Not applicable.
